# Endocarditis-Associated C3-Dominant Glomerulonephritis in a Patient With a Solitary Kidney

**DOI:** 10.7759/cureus.27675

**Published:** 2022-08-04

**Authors:** Lynda Chowdhury, Ahmed Alobaidi, Irina Lytvak

**Affiliations:** 1 Department of Internal Medicine, Methodist Dallas Medical Center, Dallas, USA; 2 Department of Pathology, Methodist Dallas Medical Center, Dallas, USA

**Keywords:** acute kidney injury, alternate pathway complement, complement mediated glomerulonephritis, mssa bacteremia, infective endocarditis, c3 glomerulonephritis

## Abstract

Infective endocarditis (IE) is still seen globally with acute kidney injuries remaining a common complication of the disease. Histological specimens often display either diffuse or focal endocapillary proliferation as well as neutrophilic infiltration in endocarditis-related renal disease. C3-dominant glomerulonephritis (C3GN) utilizes mechanisms of complement activation unique from IE-associated glomerulonephritis. In C3GN, micrographic review may reveal scattered accumulation of C3 fragments with subepithelial hump formation and mesangial electron-dense deposits that help solidify the diagnosis of this recently discovered pathological phenomenon. Herein, we summarize a clinical case of likely IE-related C3GN without hypocomplementemia in a patient with a single kidney to help compare and contrast the key elements of each process.

A 27-year-old Hispanic man with a past medical history of nephrectomy for renal donation presented to a community hospital with a high fever and altered sensorium. A serum creatinine of 6.98 mg/dL with unknown baselines, nephrotic-range proteinuria, and severe rhabdomyolysis plus methicillin-sensitive *Staphylococcus aureus* bacteremia were quickly discovered after admission. A later transesophageal echocardiogram showed a hypermobile vegetation along the anterior mitral valve leaflet confirming suspected IE. The patient’s serum C3 and C4 complement levels and antinuclear, myeloperoxidase, and proteinase-3 antibody titers were all within normal limits. A renal biopsy pursued in the etiological investigation of this non-oliguric acute kidney injury revealed a single subepithelial electron-dense deposit and granular immunofluorescent C3 staining in peripheral mesangial segments.

Dominant C3 deposition without associated immunoglobulins can result from in situ localization of bacterial antigens promoting plasmin activation to recruit neutrophils and monocytes to initiate leukocyte-mediated damage. Immunosuppressive therapies for C3GN triggering antibody-independent activation of the alternative or lectin complement pathways may be merited where disease remission becomes difficulty to achieve.

## Introduction

In infective endocarditis (IE), vegetations initially form after bacteria adhere to the endothelium of damaged or inflamed valves to form a biofilm [1]. In time, the valvular lesions mature and can lead to a disseminated infection, which can result in significant clinical symptoms and complications [1]. Acute renal failure secondary to IE-related glomerulonephritis (IE-GN) is common, and more than 80% of cases demonstrate focal, segmental, or diffusely proliferative histological patterns with infiltrating leukocytes [2]. Both C3 glomerulopathy and IE-GN often present with C3 deposits in the absence of immune complex deposition, but the mechanisms of complement pathway activation fueling the disease state differ greatly [3]. Differentiating between the two clinically remains challenging as each condition can present with hematuria, proteinuria, and normal serum C3 levels. Close follow-up to gauge renal recovery over time can help distinguish the diagnosis between these unique pathophysiological processes of IE-associated kidney injury as persistent urinary losses of blood and protein with or without hypocomplementemia will point towards a C3 glomerulopathy [3]. C3 glomerulopathy will present as either one of two known subtypes: dense deposit disease or C3-dominant glomerulonephritis (C3GN). Classification as C3GN, as discussed in the below-mentioned case, involves the deposition of scattered complement components in mesangial, subepithelial, or subendothelial locations [4,5]. Intramembranous and subepithelial electron-dense deposits may also be found [4,5]. 

This clinical scenario was previously discussed in a digital meeting presentation during the Society of Critical Care Medicine's 51^st^ Critical Care Congress, April 18-21, 2022. 

## Case presentation

A 27-year-old Hispanic man presented to the emergency department (ED) with fever and altered sensorium. The patient was capable of voicing only incomprehensible words. No information related to his past medical, family, surgical, or social histories could be obtained on arrival. An oral temperature of 39.3°C was measured with a blood pressure reading of 132/77 mm Hg, a heart rate of 125 beats per minute, and an oxygen saturation of 89% on room air. His oxygen saturation later improved to 97% with two liters of supplemental oxygen. Given his altered mental state, he was unable to follow commands; nonetheless, no focal neurologic deficits were apparent.

**Figure 1:**
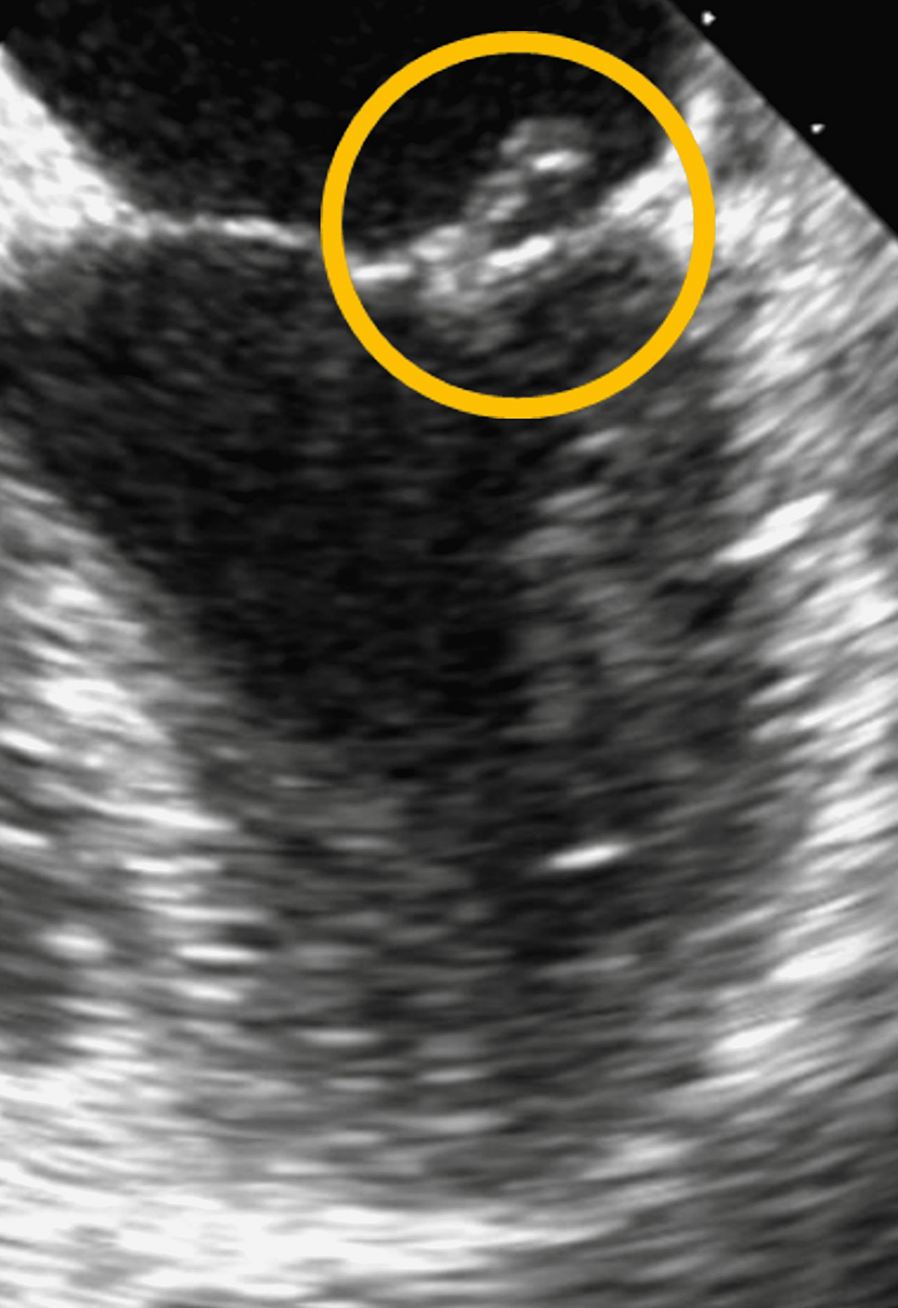
A transesophageal echocardiogram performed early during the patient’s hospitalization showed a hypermobile vegetation along the anterior mitral valve leaflet (circled) confirming suspected infective endocarditis.

Laboratory tests notably showed a serum white blood cell count of 15,100 cells/µL with a neutrophilic predominance of 88.9% and a platelet count of 55,000 cells/µL without bleeding. Hemoglobin and hematocrit levels of 13.8 g/dL and 37% respectively and a mean corpuscular volume of 90.2 fL were within normal limits. A comprehensive metabolic panel ordered on admission revealed a serum bicarbonate level of 21 mmol/L, a glucose level of 178 mg/dL, a blood urea nitrogen (BUN) level of 65 mg/dL, a serum creatinine of 6.98 mg/dL, a serum calcium level of 8.2 mg/dL, a total bilirubin level of 4.9 mg/dL, and an aspartate aminotransferase level of 141 U/L. Serum sodium, potassium, chloride, albumin, total protein, alkaline phosphatase, and alanine aminotransferase levels were all within normal limits. Urinalysis revealed a large amount of blood with urine protein levels of 534 mg/dL, moderate levels of leukocyte esterase, urine white blood cell counts of 50-100 cells/high-power field, and urine red blood cell counts of two to five cells/high-power field. No urinary bilirubin, ketones, glucose, or nitrites were detected. A spot urine protein-to-creatinine ratio was 4.36. Blood cultures collected on admission later resulted in the growth of methicillin-sensitive* Staphylococcus aureus* (MSSA). Incidentally, urine cultures grew this same organism suggesting a urinary source for the bacteremia. A transthoracic echocardiogram demonstrated normal left ventricular chamber size, wall motion, and contractility with an estimated ejection fraction of 60-65%, and global right systolic function was preserved. A 1.2-cm by 0.6-cm vegetation was spotted on the anterior mitral valve leaflet with mild-to-moderate mitral regurgitation, no mitral valve stenosis, and mild-to-moderate tricuspid regurgitation. No pericardial effusion was present. A transesophageal echocardiogram showed the same hypermobile vegetation along the anterior mitral valve leaflet confirming suspected IE (Figure 1). A urine drug screen revealed the presence of both amphetamine and cocaine metabolites. Serum creatine phosphokinase (CK) levels peaked at 13,400 units/L five days after arrival but were initially measured at 4,012 units/L. The patient’s serum C3 and C4 complement levels and antinuclear, myeloperoxidase, proteinase-3 antibody titers were all within normal limits. No acute hepatitis A, B, or C infections were identified, and he was negative for HIV. A peripheral smear revealed a bicytopenia of the red cells and platelets without red cell schistocytosis and an elevated white cell count consisting primarily of mature neutrophils plus toxic granulations, Dohle bodies, and vacuolations concerning for infection. Haptoglobin and lactate dehydrogenase (LDH) levels were measured at 492 mg/dL and 1296 U/L respectively.

A renal Doppler ultrasound revealed the absence of a right kidney. The patient’s brother later disclosed that the patient had donated the organ to him eight years prior. No abnormalities of the left kidney or the bladder were cited. The patient's family members, upon further questioning, were unable to name the hospital where the living-donor renal transplant had occurred or the providers who would have performed a medical evaluation of the patient prior to his attempted donation. The patient also did not have a primary-care provider nor were he or his family members ever able to name any doctors visited in the past that might possess any clinical records that could be reviewed to assess baseline kidney function or any other pertinent medical information. 

A nephrologist was consulted soon after the patient was admitted to the intensive care unit. Initial differentials included renal thrombotic microangiopathy given notable thrombocytopenia, fever, and encephalopathy with acute kidney injury (AKI), but no schistocytes were seen on peripheral smear. Other diagnoses like a cocaine-associated AKI, rhabdomyolysis-induced AKI, acute interstitial nephritis secondary to a disseminated staphylococcal infection, ischemic acute tubular necrosis related to sepsis, immune complex-mediated glomerulonephritis, septic renal emboli with infarction, and even secondary or acquired AA amyloidosis with a history of intravenous (IV) and subcutaneous drug use were also entertained. 

No renal replacement therapies were indicated at first as the patient was able to produce adequate urine while remaining relatively euvolemic with only mild electrolyte imbalances. He was started on high-rate IV fluids and managed supportively. However, over the next five days after admission, the patient's serum creatinine and BUN levels continued to rise steadily up to a peak of 7.20 mg/dL and 109 mg/dL respectively. He underwent two hemodialysis sessions in the first four days of his hospital stay given both persistent uremia and continued encephalopathy. His platelet counts began to gradually improve from 55,000 cells/µL to 123,000 cells/µL within five days after admission and later stabilized around 161,000 cells/µL by the time of discharge. As his renal function remained poor with no sign of improvement, the nephrologist began to consider pursuing a percutaneous kidney biopsy to better establish an exact diagnosis among a large number of possibilities, especially as multiple concomitant processes were suspected to be contributing to the patient's presentation. Given that no assessments of the patient's baseline renal function could be made as records of said clinical information remained unavailable, a biopsy was also deemed useful in assessing the degree of active or chronic changes to better determine prognosis and guide appropriate treatment. To minimize all potential complications of this high-risk procedure in a patient with a solitary native kidney, the nephrologist recommended waiting to perform the biopsy until after the patient's platelet count normalized and his BUN levels had dropped after dialysis. The patient verified that he had not been taking any antiplatelet or antithrombotic drugs in the preceding two weeks before his planned biopsy, and all chemical venous thromboembolism prophylaxis was stopped 24 hours before the procedure. 

**Figure 2:**
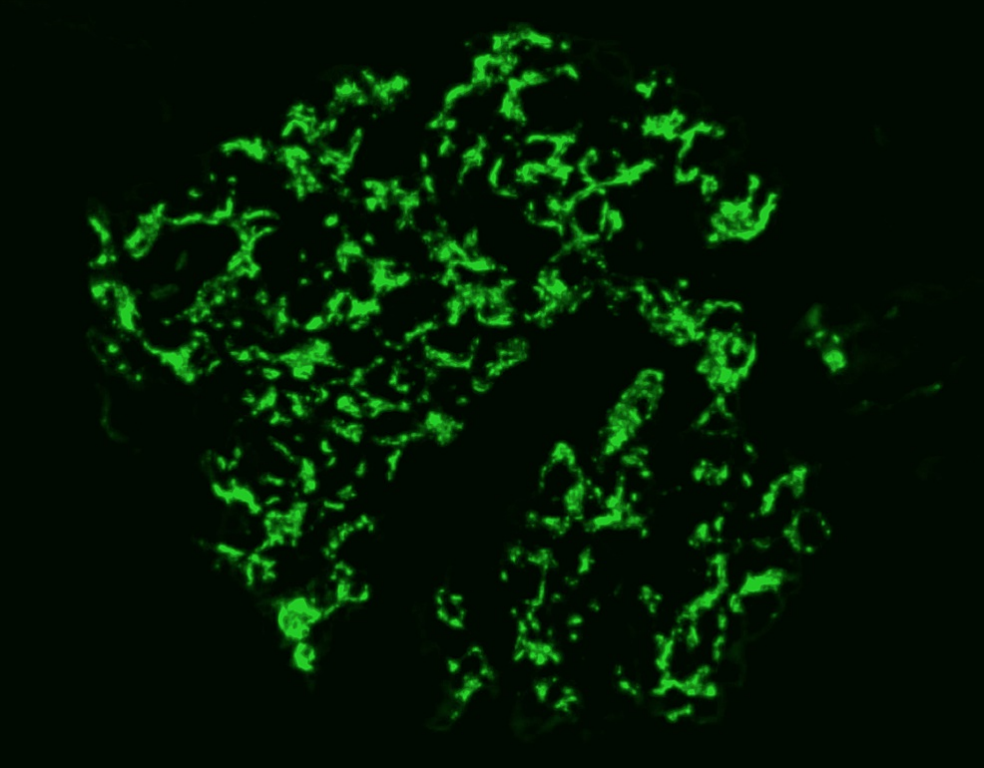
An immunofluorescence microphotograph showing strong glomerular C3 staining.

**Figure 3:**
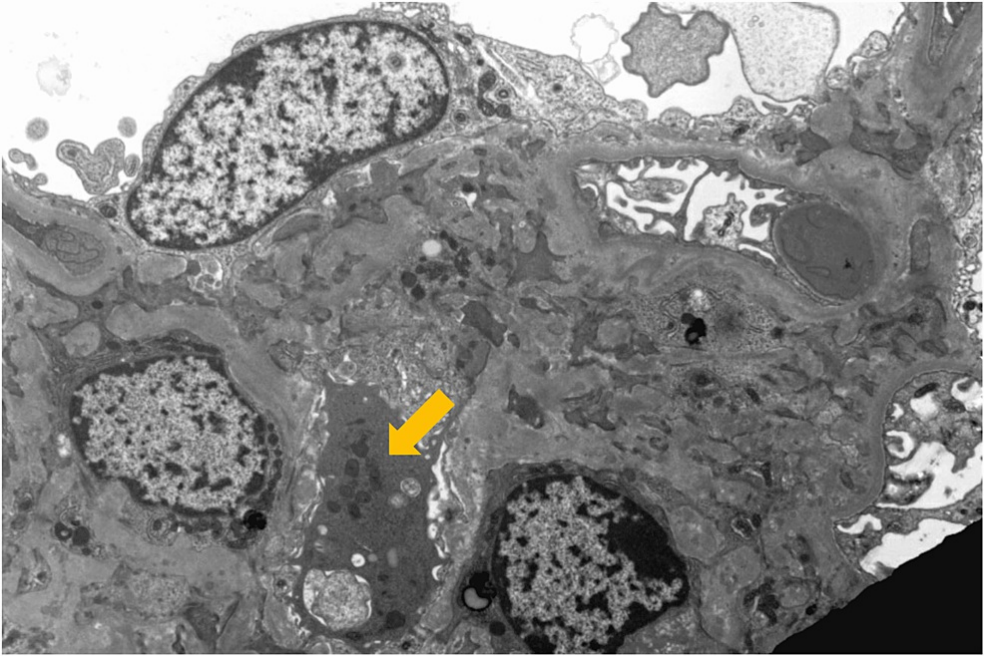
An electron microphotograph showing a large subepithelial deposit or “hump” (arrow) near the bottom-left corner of the image.

Four core renal biopsies were collected. Among these samples, 17 glomeruli were microscopically visualized, and five of them showed globally sclerotic and obliterated capillary loops. Additionally, immunohistochemical staining for myoglobin within the tubular lumens was positive. No glomerular arterial thrombosis or vasculitis were seen. Mesangial and segmental immunofluorescent C3 staining reflected C3 deposition peripherally along cell borders (Figure 2). No immunoglobulins or C1q deposits were cited. Electron-dense deposits and a single, large subepithelial hump were also visualized (Figure 3).

The patient’s anterior mitral leaflet vegetation was operatively resected, and a mitral valve annuloplasty was performed. He further concomitantly completed a six-week course of intravenous cefazolin. Though some renal function recovery was cited during the patient’s admission, his serum creatinine stabilized between 2.80 and 3.05 mg/dL with an estimated glomerular filtration rate of 24.8 to 27.3 mL/minute/1.73 m^2^. He never received any immunosuppressive treatments during this hospitalization. Unfortunately, he was lost to follow-up after his discharge, so any additional renal recovery remained undetermined.

## Discussion

As a key mediator of innate immunity, the complement cascade consists of three pathways. Each pathway is activated by differing mechanistic triggers leading to the ultimate generation of pro-inflammatory mediators, the formation of the membrane attack complex (MAC) to lyse pathogenic organisms, and the enhancement of phagocytic processes via opsonization [6]. No reductions in C3 or C4 levels indicate that the alternative complement pathway was likely activated in this case of IE-related C3GN. The alternative pathway remains continually active in the body and generates C3b that can bind to both host cells and foreign microbes without discrimination [6]. C3 binds with activator complement factor B when attacking a bacterium that will later be cleaved by factor D. Subsequently, it forms alternative C3 convertase leading to an amplification loop that cleaves exponentially more C3 to form C5 convertase [6]. This then leads to the formation of the MAC and allows C9 to form a pore in the cell membrane triggering apoptosis and ultimate cell death.

Dense capillary wall and mesangial C3 staining will often accompany histological patterns of IE-GN and C3GN [2]. In C3GN, prominent C3 glomerular detection in the distinct absence of immunoglobulin, C1q, or C4d depositions highlight activation of the alternative complement pathway [5,7,8]. C3GN is readily distinguished from immune-complex glomerulonephritis in that no classical pathway proteins save C3 are present in any kidney deposits studied [2]. C3d fragments may also be identified in biopsy specimens with evidence of acute ischemic or tubular injuries as immune responses to foreign antigens are altered in the cross-talk between complement activation and the adaptive immune system [2]. 

Treating the underlying infection in IE-GN leads to near-total resolution of renal disease via supportive care alone in time [3]. C3GN, however, may not as readily resolve given poor prognoses leading to end-stage renal disease development over 10 years. In addition, no robust guidelines exist to aid in steering treatment directions [3]. Because of loss to follow-up, the patient could not be monitored via regular visits to see if any recurrent disease appeared in the absence of anti-inflammatory drug therapies. Immunosuppressive agents (e.g., mycophenolate mofetil or prednisone) and targeted therapies against components of the complement pathway (e.g., eculizumab) have both been shown to induce remission in cases of moderate inflammatory disease [3,9]. Given the paucity of data for severe illness from this disease process, there remains no consensus on long-term treatment [3].

Performing a percutaneous renal biopsy on a solitary native kidney is relatively contraindicated as severe post-biopsy complications may lead to a nephrectomy that would result in the loss of the patient's sole functioning kidney. However, percutaneous kidney biopsies have historically had low overall complication rates. Among 19,459 biopsies performed between 1951 to 1990, complications were seen in 2.1-10.8% of cases, and only 13 nephrectomies were required because of these complications [10]. As the patient's kidney function failed to improve and information related to his renal baselines were unknown given that no prior medical records could be obtained for review, the benefits of pursuing a percutaneous biopsy in a single kidney outweighed the general risks according to the expert opinion of the nephrologist following this case. 

## Conclusions

Both IE-GN and C3GN can easily be triggered by severe disseminated infections and share many overlapping histopathological features. Each disease process can exhibit C3 deposition without immune-complex formation and endocapillary proliferation with subepithelial humps. Though a highly-suspected diagnosis given biopsy findings in this case, investigations to assess repeat hematuria or proteinuria at least two or three months after infection as well as genetic studies to note alternative pathway dysfunction secondary to mutations in complement genes would have added more support to the diagnosis of C3GN made in this case presentation. Limited knowledge presently undergirds clinical treatment recommendations as few studies comparing therapeutic options in diagnosed C3GN currently exist. In time, nevertheless, one expects that advances in diagnostic techniques coupled with further discoveries related to complement-mediated glomerular disorders will advance the clinician's abilities to both identify and treat this disease process. 
